# Global Motion Percept Mediated through Integration of Barber Poles Presented in Bilateral Visual Hemifields

**DOI:** 10.1371/journal.pone.0074032

**Published:** 2013-08-29

**Authors:** Li-Ting Huang, Alice M. K. Wong, Carl P. C. Chen, Wei-Han Chang, Ju-Wen Cheng, Yu-Ru Lin, Yu-Cheng Pei

**Affiliations:** 1 Department of Physical Medicine and Rehabilitation, Chang Gung Memorial Hospital at Linkou, Taoyuan, Taiwan; 2 School of Medicine, Chang Gung University, Taoyuan, Taiwan; 3 Healthy Aging Research Center, Chang Gung University, Taoyuan, Taiwan; University of Regensburg, Germany

## Abstract

How is motion information that has been obtained through multiple viewing apertures integrated to form a global motion percept? We investigated the mechanisms of motion integration across apertures in two hemifields by presenting gratings through two rectangles (that form the dual barber poles) and recording the perceived direction of motion by human observers. To this end, we presented dual barber poles in conditions with various inter-component distances between the apertures and evaluated the degree to which the hemifield information was integrated by measuring the magnitude of the perceived barber pole illusion. Surprisingly, when the inter-component distance between the two apertures was short, the perceived direction of motion of the dual barber poles was similar to that of a single barber pole formed by the concatenation of the two component barber poles, indicating motion integration is achieved through a simple concatenation mechanism. We then presented dual barber poles in which the motion and contour properties of the two component barber poles differed to characterize the constraints underlying cross-hemifield integration. We found that integration is achieved only when phase, speed, wavelength, temporal frequency, and duty cycle are identical in the two barber poles, but can remain robust when the contrast of the two component barber poles differs substantially. We concluded that a motion stimulus presented in bilateral hemifields tends to be integrated to yield a global percept with a substantial tolerance for spatial distance and contrast difference.

## Introduction

A common problem faced by the neural system is integrating spatially distributed information to infer a global percept. Researchers proposing Gestalt psychology observed that we tend to link multiple components together to create a global pattern percept [1,2]. However, Gori and Spillmann found that grouping and segregation are not symmetrical, as integration occurs under more strict constraints [3]. It then remains unclear how visual motion emanating from spatially segregated areas forms the global motion. Specifically, we seek to understand how two component objects presented at each side of the visual field (hemifield) are perceived as a whole. It is interesting to characterize the basic constraints of integration, such as the spatial arrangement, shape, and direction of motion of each of the component objects, as well as to understand the rules that determine the perceived velocity (direction and speed). For example, one might ask whether one object seen through multiple apertures is regarded as a single entity or as multiple individual objects. Also, it remains unclear whether the human brain can construct a single global motion percept even when two component objects are presented far apart.

Integration and segregation are two processes that are complementary to each other [4–6]. For example, when confronted with moving images, the human brain has to decide whether the motion signals arise from a single object or from multiple objects. Figure ground segregation is a process that groups contours together, which is fundamental to the neural system for integrating information. As for continuously changing stimuli, alternative figure-ground organizations are resolved via low-level, dynamical competition [7]. Tommasi and Vallortigara [8] investigated the effect of figure ground segregation on the modulation of perceived direction by presenting a circular drifting grating bisected by a vertical non-transparent bar. The results showed that the human brain integrates the split-half portions to reconstruct a global grating motion. Shimojo et al. reported that gratings presented through spatially discrete component apertures can be “grouped” together to yield a global percept [9]. Though the exact mechanisms underlying this integration are still to be determined, this kind of integration was suggested to be achieved by the mechanism of amodal completion [10–12], the processing to perceive the whole of an object when only parts of it are “seen” by the sensory sheet [13].

We hypothesized that the motion processing property provides a unique opportunity to qualify the integration properties. The aperture problem of motion processing is a well-studied example of the integration process [14–22]. When a moving bar is observed through a circular aperture, no matter where it moves forward, the perceived velocity is always perpendicular to its orientation, implying that the local information could be insufficient to infer the velocity of the global motion. In order to infer a veridical percept of an object’s velocity, it is necessary to integrate motion information across multiple stimulus contours that differ in orientation [23,24] or to rely on terminators—such as end points, corners, and intersections—whose velocities are unambiguous. The aperture problem implies that neurons with small receptive fields could yield inaccurate velocity inferences [25–32]. However, recent studies suggest that terminator information could be encoded in the candidate neurons in the primary visual cortex named end-stopped neurons [33–39].

Guilford found that a barber pole, constructed by the presentation of a drifting grating through a rectangular aperture, yields a visual motion illusion which can characterize how motion integration is processed in the human brain [14,40–42]. The perceived direction of motion of a barber pole is biased towards the long axis of the aperture, a phenomenon dubbed “the barber pole illusion” [43–47]. Psychophysical and neurophysiological studies have suggested that the terminators are used to determine the direction of motion of a barber pole [38,48–50]. Specifically, perceived direction is biased towards the long axis of the aperture because the long axis is comprised of more terminators than the short axis.

In the present study, we presented dual barber poles which consisted of two component barber poles placed symmetrically in two hemifields and then recorded the perceived direction of motion in human observers. By analyzing the strength of the barber pole illusion induced by the dual barber poles, we could gauge the strength of motion integration across the hemifields. We varied the distance between the component barber poles to characterize the spatial constraints of integration, and then we presented dual barber poles in which the motion and contour properties of the two component barber poles differed to characterize the motion and contour constraints underlying integration. The results indicated that motion information presented in the bilateral hemifields tended to be integrated to yield a global percept with a substantial tolerance of spatial distance and contrast difference between the two component barber poles.

## Methods and Results

### Human subjects

Five healthy naive subjects (2 men, 3 women, ranging from 20 to 37 years old) participated in all the experiments in the present study. All subjects reported normal corrected vision and no history of central nervous system disease. The corrected vision of all subjects was assessed by the Snellen chart, and all subjects showed visual acuity equal to or better than 1.0. Written informed consent was obtained from each subject. All testing procedures were performed in compliance with the policies and procedures of the Chang Gung Medical Foundation Institutional Review Board. The Chang Gung Medical Foundation Institutional Review Board specifically approved this study. Subjects were paid for their participation.

### The visual stimuli

#### Barber pole

**Figure 1:**
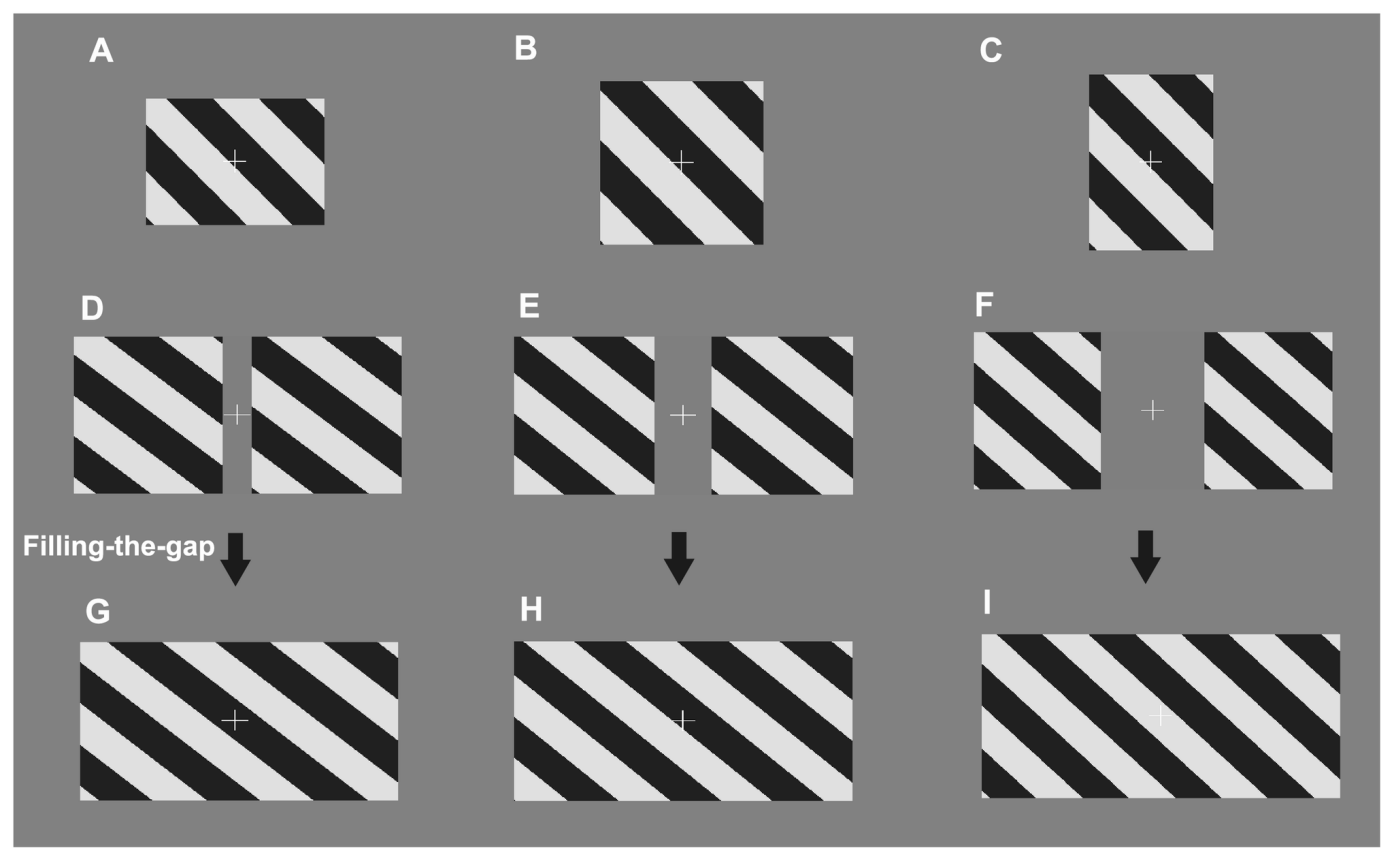
Examples of visual stimuli and illustrations of the filling-the-gap hypothesis. A–C. Single barber pole with aspect ratios (height: width) of (A) 1:1 (1.19°/1.19°), (B) 1:1.41 (1°/1.41°), and (C) 1.41:1 (1.41°/1°), and a fixed aperture area of 1.41 (°)^2^. D–I. To test whether the perceived direction of the dual barber poles is mediated through the filling-the-gap hypothesis, we presented a series of completed barber poles (as shown in G, H, and I) constructed by filling the grating into the inter-component space of the dual barber poles in D, E, and F, respectively.

A barber pole was constructed by presenting a drifting square-wave grating within a rectangular aperture (example barber poles are shown in Figure 1A and 1C); only pixels within the aperture were active. The directions of the movements were 45^°^, 135^°^, 225^°^, or 315^°^, which were ± 45° relative to the orientation of the long axis of the aperture. Unless otherwise specified in the following text, the wavelength (stripe-to-stripe distance) of the drifting square-wave gratings constituting the barber poles was 0.5°, their duty cycle 50%, their speed 10°/s, and their contrast 75% (computed using the Michelson formula). The luminance levels of the grating stripes were 4.5 cd/m^2^ and 31.7 cd/m^2^, respectively, against a background of 18.1 cd/m^2^. We used this wavelength and duty cycle because it yielded a clear percept of the barber pole illusion as determined in preliminary experiments and by other researchers [20]. The strength of the barber pole illusion was characterized by the degree to which the perceived direction was biased toward the long axis of the aperture.

#### Dual barber poles

Dual barber poles consist of two component barber poles symmetrically presented in bilateral visual hemifields, separated by an inter-component distance (example dual barber poles shown in Figure 1D–F). Each component barber pole has an aspect ratio of 1: 1.41 (width: height: = 1^°^: $\sqrt{2}$ ^°^). The rationale for choosing this aspect ratio is that the side-by-side concatenation of two component barber poles yields a single horizontal barber pole with an aspect ratio of 1.41:1 (width: height: = 2^°^: $\sqrt{2}$ ^°^), a design that could balance the effects between the component barber poles and the global barber pole ensemble. Unless otherwise specified, the phase of the two component barber poles was identical.

### Procedures

#### Task structure

**Figure 2:**
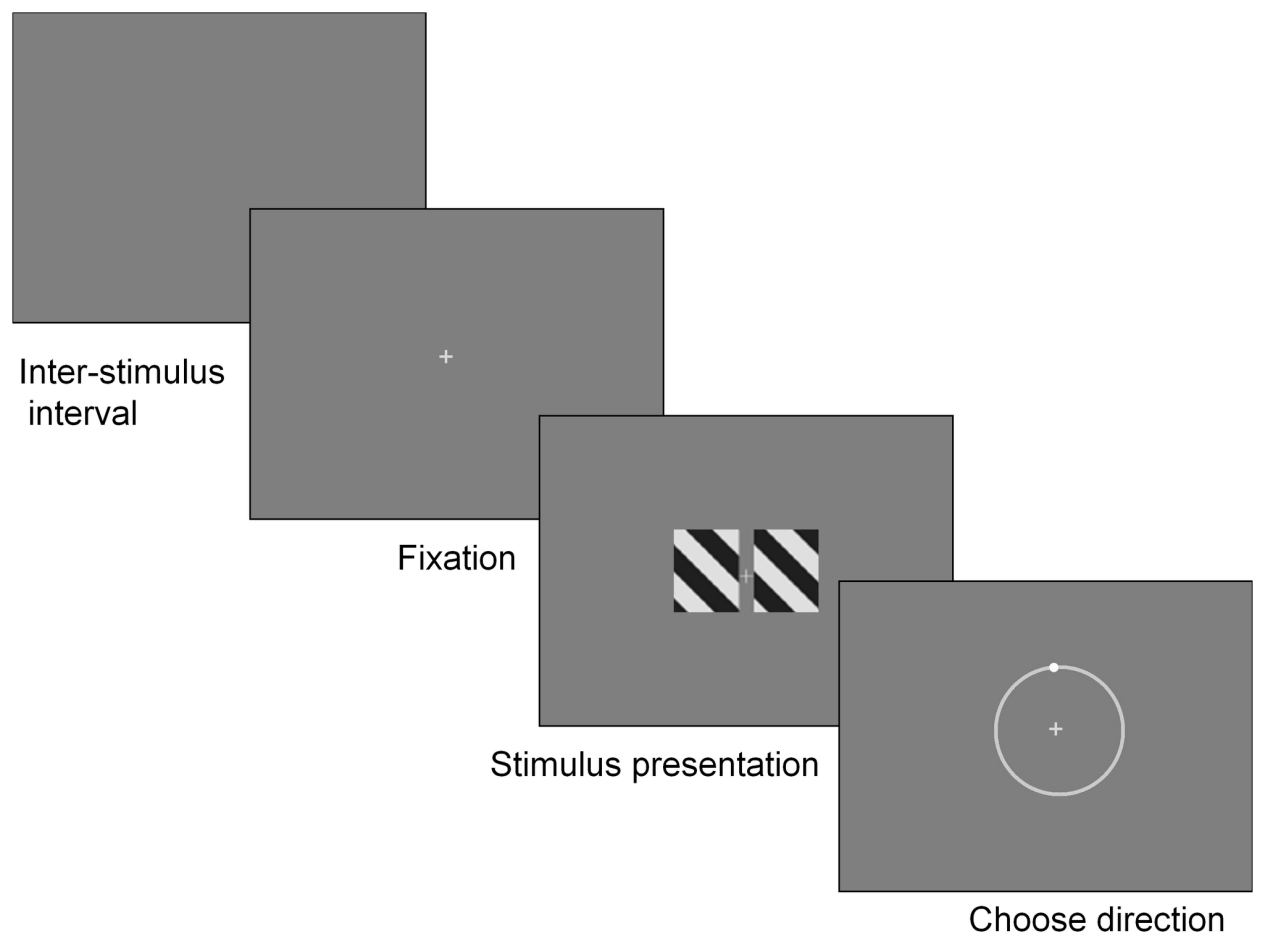
The task structure of direction identification to dual barber pole stimuli. A cross with a width of 0.17° first appeared at the center of the display for 200ms and the subject fixated on the cross. The dual barber poles were displayed for 1 s and the subject reported the perceived direction of motion by mouse clicking on a sliding cursor along a round track.

Subjects viewed visual stimuli binocularly at a distance 96 cm in front of a monitor in a dark room. The stimuli were generated by a personal computer and displayed on a CRT monitor (ViewSonic E70, Model No: VCDTS21569-3P, ViewSonic Corporation, Walnut, CA) with a resolution of 1024*768, a pixel size of 0.02°, and a refresh rate of 60 Hz. The software was Matlab (Mathworks, Natick, MA) with Psychophysics Toolbox [51]. On each trial, a cross of 0.17° first appeared at the center of the display for 200ms and the subject was asked to fixate on the cross throughout the stimulus period (Figure 2). The visual stimulus was then displayed for 1s and, after the stimulus period, the subject reported the perceived direction of motion by mouse clicking one sliding cursor along a round track on the computer screen. The round track was divided from 0° to 359° in 1° steps. We developed a task structure that could allow the subject to report two types of motion percept to the dual barber pole stimulus. In one type of percept dubbed the “global percept”, the subject experienced the two component barber poles moving at one velocity (speed and direction) and reported the perceived global direction using left mouse click. In another type of percept dubbed the “individual percept”, the subject perceived two motion velocities corresponding to the velocity of each of the two component barber poles. The subject reported this type of percept by clicking the right mouse button. There was a 200-ms interval between the subject’s response and the subsequent stimulus. Each type of stimulus was presented at least 5 times in pseudo-random order. Each protocol was split into 5 blocks to allow the subject a break.

### Data Analysis

The *perceptual bias*, which gauged the magnitude of the barber pole illusion, was expressed as the deviation of the perceived direction from the grating’s direction of motion and was defined as positive when it deviated horizontally. In other words, a positive perceptual bias indicated that the perceived direction was biased toward horizontal directions and a negative perceptual bias indicated that the perceived direction was biased toward vertical directions.

As mentioned in the task structure section, the subject could either perceive a global motion coherently moving at one velocity (global percept) or two motion velocities corresponding to the velocity of each of the two component barber poles (individual percept). We then computed the *global probability* as the proportion of trials where the subject reported the “global percept” by choosing the perceived global direction, instead of reporting the “individual percept” by only clicking the right mouse button.

The perceptual bias among repetitions was averaged using the vector average method and the data was represented as mean ± standard error of mean. The comparisons among conditions were analyzed using repeated-measures ANOVA.

#### Experiment 1: single barber pole with various aspect ratios

We presented single barber poles with various aspect ratios and with a fixed aperture area to gauge the magnitude of the barber pole illusion in each subject. The three different aspect ratios (height/width) were 1:1.41 (1°/1.41°), 1:1 (1.19°/1.19°), and 1.41:1 (1.41°/1°) and their aperture area was fixed as 1.41(°)^2^ (Figure 1A–C).

**Figure 3:**
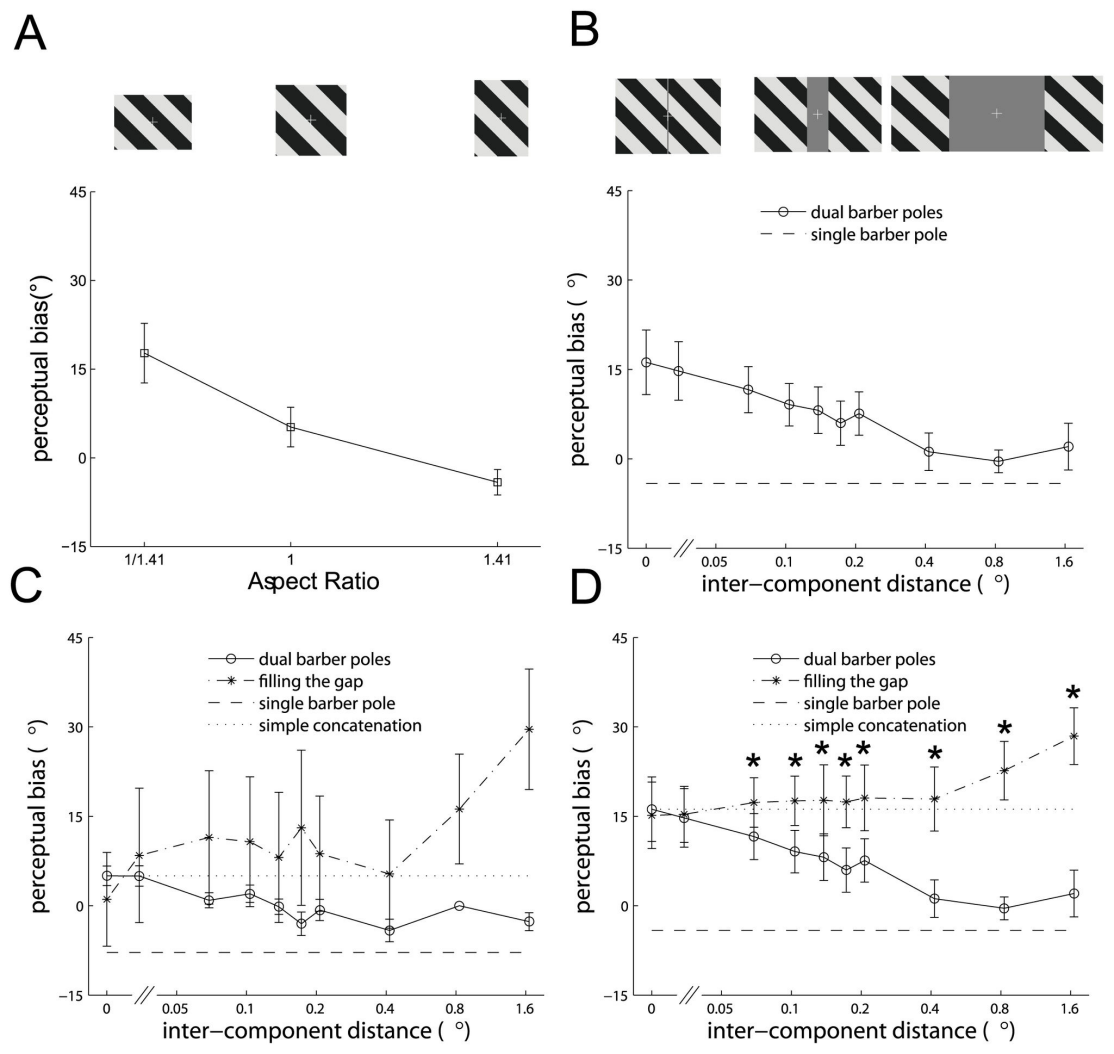
The perceived directions of single and dual barber poles and the predictions of the perceived directions of the dual barber poles based on the filling-the-gap and simple concatenation hypotheses. A. The mean perceptual bias toward the horizontal direction as a function of the aspect ratio induced by single barber poles illustrated in Figure 1A–C. The three barber poles with aspect ratios (height/width) of 1:1.41 (1°/1.41°), 1:1 (1.19°/1.19°), and 1.41:1 (1.41°/1°) are illustrated in the upper panel. The error bars indicate standard error of mean. B The mean perceptual bias toward the horizontal direction induced by the dual barber poles as a function of inter-component distance. Examples of the dual barber poles with inter-component distances of 0.03°, 0.41°, and 1.66° are illustrated in the upper panel. The perceptual bias to dual barber poles (circle trace) peaked when the inter-component distance was zero (yielding a single horizontal barber pole) and, as the inter-component distance increased, gradually decreased to approach the perceived direction of the single component barber pole. The dashed line indicates the perceptual bias induced by the single component barber pole (with an aspect ratio of 1.41:1) shown in (A). C–D. The perceptual bias to the dual barber poles at various inter-component distances (circle trace), the predictions made by the filling-the-gap (star trace) and simple concatenation hypotheses (dotted line), and the perceptual bias induced by the single component barber pole (dashed line), observed in an example subject (C) and in the mean across subjects (D) (* symbols indicate significant difference between the dual barber pole and completed barber pole, *p* < 0.05 using repeated-measures ANOVA).

Figure 3A showed the perceptual bias, the degree to which the perceived direction deviated from the direction of the grating, to the single barber pole with various aspect ratios. The horizontal barber pole with a width-to-height aspect of 1.41:1 elicited a mean bias of 17.7° toward the horizontal axis and the vertical barber pole with a width-to-height aspect of 1: 1.41 elicited 4.1° bias toward the vertical axis, indicating the existence of the barber pole illusion in these subjects. The square grating with an aperture of 1:1 induced a 5.2° bias toward the horizontal axis, implying that the subjects tended to perceive a grating moving more horizontally. The perceptual bias differed significantly among the three conditions (F(2,8) = 34.4, *p* < 0.001).

#### Experiment 2: the effect of inter-component distance on the strength of cross-hemifield integration

We presented dual barber poles with different inter-component distances to measure the degree to which the inter-component distance affected the strength of motion integration across the visual hemifields. The distance between the two component barber poles was 0°, 0.03°, 0.07°, 0.10°, 0.14°, 0.17°, 0.21°, 0.41°, 0.83°, or 1.66° (example of the dual barber poles shown in Figure 3B upper panel).

For dual barber poles with various inter-component distances, the probability of global percept, the proportion of trials the subject reported a global velocity, was 1.0 (100%) in all conditions and in all subjects, indicating these dual barber poles always elicit a single global percept; the perceptual bias peaked when the inter-component distance was zero, yielding a single horizontal barber pole with a width-to-height aspect ratio of 1.41:1 (Figure 3B circle trace). As the inter-component distance increased, the bias gradually decreased (F(9,36) = 8.7, *p* < 0.001) and then approached the perceived direction of the single vertical component barber pole measured in Experiment 1 (Figure 3B dashed line). The change in the magnitude of the barber pole illusion as a function of the inter-component distance indicated that the inter-component distance determines the strength of cross-hemifield motion integration. Specifically, when the component barber poles are close to each other, the visual system considers the two component apertures as a whole and computes the “global” perceived direction of motion accordingly.

#### Experiment 3: the filling-the-gap hypothesis

We proposed two cross-hemifield motion integration mechanisms that might account for the results observed in the dual barber poles with various inter-component distances (Figure 3B) in Experiment 2: the filling-the-gap hypothesis and the simple concatenation hypothesis. In the filling-the-gap hypothesis, the dual barber poles were perceived as a single barber pole constructed by filling the inter-component space with grating, which is a type of amodal completion (Figure 1G–I). In the simple concatenation hypothesis, the dual barber poles are perceived as a single horizontal barber pole constructed simply by annexing the two component barber poles side-by-side. The simple concatenation hypothesis predicts perceptual bias as a constant (equal to the results when the inter-component distance is 0°) among all the inter-component distance conditions.

We presented completed barber poles constructed by filling the area between the component barber poles in Experiment 2 with grating, which was compatible with the filling-the-gap mechanism and measured the perceived bias induced by them (examples shown in Figure 1G–I). The width of the completed barber pole, corresponding to each of the dual barber poles, was 2°, 2.03°, 2.07°, 2.10°, 2.14°, 2.17°, 2.21°, 2.41°, 2.83°, or 3.66°, and its height was 1.41°.

Perceptual bias to the completed barber poles, reflecting the filling-the-gap predictions, monotonically increased as the inter-component distance increased (star trace, example subject in Figure 3C and the mean across subjects in Figure 3D (F(9,36) = 7.45, *p* < 0.001). This finding was incompatible with their perceived directions to the dual barber poles (circle trace in Figure 3C-D) (interaction effect between barber pole type and inter-component distance, F(9,36) = 13.96, *p* < 0.001). As can be seen in Figure 3C-D, the perceptual bias was identical to that of the annexed dual barber poles (dotted line) when the inter-component distance was small (post-hoc analysis, *p* > 0.05 when the width of the completed barber pole was 2° and 2.03°) and gradually shifted to that of the single component barber pole (dashed line) as the inter-component distance increased (post-hoc analysis, *p* < 0.05 when the width of the completed barber-pole was **e**qual and more than 2.07°), suggesting that cross-hemifield integration follows the simple concatenation mechanism when the inter-component distance is small, and the strength of integration faded away when the two components are far apart.

The strength of cross-hemifield integration gradually decreased instead of abruptly dropping as the inter-component distance increased, implying that cross-hemifield integration did not follow the winner-take-all mechanism. In the following experiments, we characterized the degree to which contour and motion factors, including wavelength, temporal frequency, speed, duty cycle, phase, and contrast affected the strength of cross-hemifield integration.

#### Experiment 4: Discrepancy in wavelength between the component barber poles

**Figure 4:**
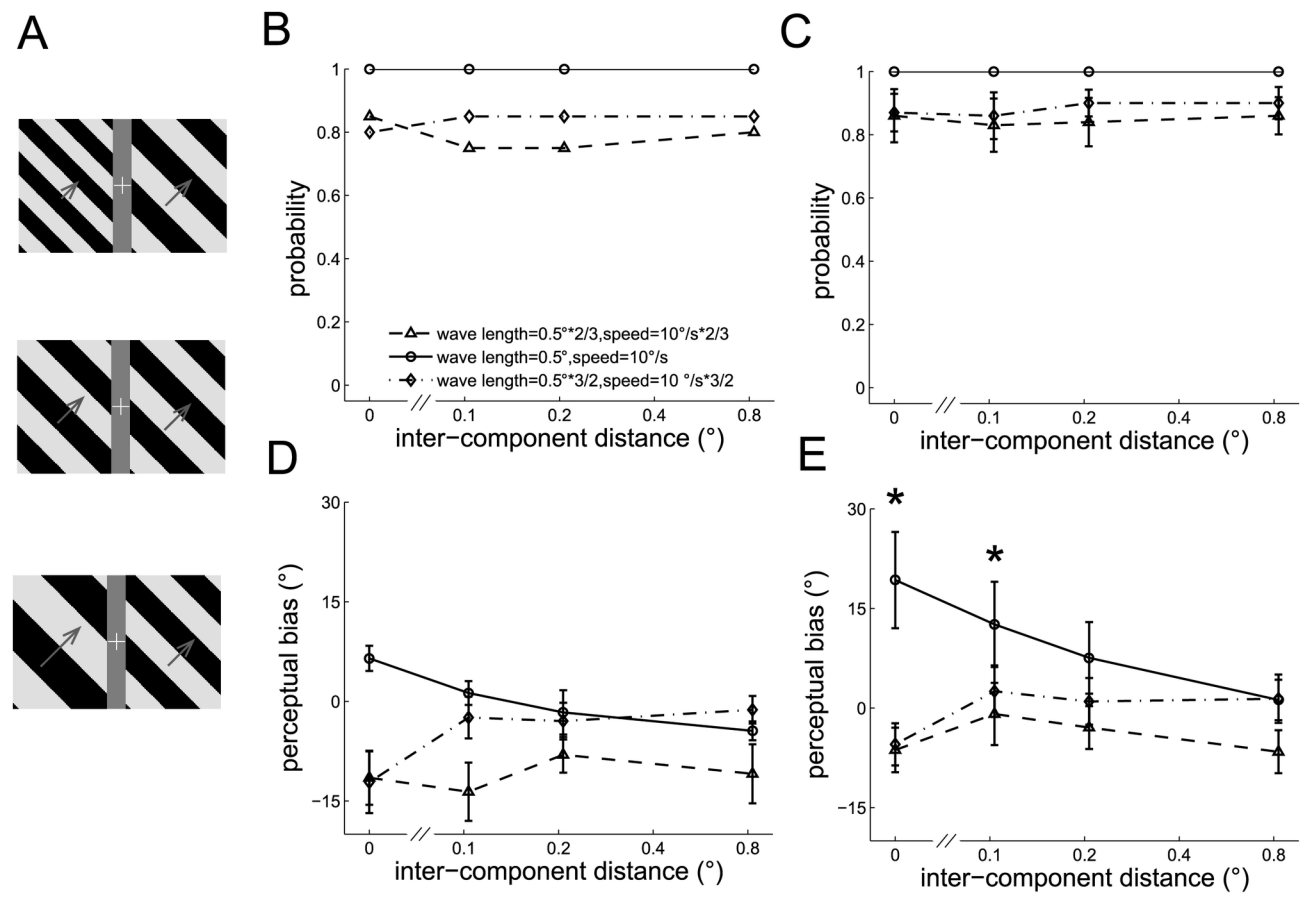
The perceived directions of the dual barber poles in which the wavelength of the two component barber poles differed. A. Three examples of dual barber poles (inter-component distance = 0.21°) with the wavelength of the left component barber pole 2/3 times (upper panel), equal (middle panel), or 3/2 times (lower panel) that of the right component barber pole. The length of the arrows indicates the speed of the drifting grating. B–C. The probability the subject perceived a global percept instead of two individual component barber poles moving at different velocities observed in an example subject (B) and in the mean across subjects (C). The standard dual barber poles always elicited a global percept and the dual barber poles in which the wavelength differed in the component barber poles still tended to elicit a global percept. D–E. The perceptual bias observed in an example subject (D) and in the mean across subjects (E) decreased when the wavelength differed in the component barber poles and the inter-component distance was small (* symbols indicate the inter-component distance in which the perceptual bias differed among the three temporal frequency conditions, *p* < 0.05).

In order to characterize the spatial-temporal constraints that determine the strength of cross-hemifield motion integration, we varied the wavelength in the left component barber pole while keeping its temporal frequency (20 Hz) the same. The parameters of the right component barber pole in this and the remaining experiments were identical to those delineated in Experiment 3. The wavelength of the left component barber pole was 0.33° (0.5°*2/3), 0.5°, or 0.75° (0.5°*3/2) and that of the right component barber pole was 0.5°. The speed, which is the product of wavelength and temporal frequency, changed proportionally as we altered the wavelength. In other words, the speed of the left component barber-pole was 2/3, 1, or 3/2 times of the speed of the right component barber poles (10°/s), respectively. The inter-component distance was 0°, 0.1°, 0.2°, or 0.8° (Figure 4A). The other parameters were identical to those used in Experiment 3.

The standard dual barber poles almost always elicit a global percept (global probability is close to 1) and the dual barber poles, in which the wavelength and corresponding speed in the component barber poles differed, still tended to elicit a global percept (global probability all > 0.8) (example subject in Figure 4B and the mean across subjects in Figure 4C). Most importantly, the perceptual bias was weakened when the wavelength differed between the component barber poles (wavelength main effect, F(2,8) = 18.7, *p* = 0.001; interaction effect between the wavelength and inter-component distance, F(6,24) = 11.3, *p* < 0.001). Post-hoc analysis showed that the perceptual bias decreased when the wavelength differed in the component barber poles and the inter-component distance was small (*p* < 0.05 when the inter-component distance was 0° and 0.1°) (example subject in Figure 4D and the mean across subjects in Figure 4E), indicating that cross-hemifield integration is weakened by the discrepancy in wavelengths between the component barber poles even though their temporal frequency is still identical. The discrepancy in wavelength did not affect the perceptual bias when the inter-component distance was long (*p* > 0.05 when the inter-component distance was 0.2° and 0.8°).

#### Experiment 5: Discrepancy in temporal frequency between the component barber poles

We then varied the temporal frequency in the left component barber pole while keeping its wavelength the same to characterize the effect of temporal frequency on the strength of cross-hemifield motion integration. The temporal frequencies of the left component barber pole were 20 Hz*2/3, 20 Hz, and 20 Hz*3/2 and the right component barber pole was 20 Hz. The wavelength was fixed at 0.5°. Correspondingly, the speed of the left component barber pole was 2/3, 1, or 3/2 times the speed of the right component barber poles (10°/s), respectively.

**Figure 5:**
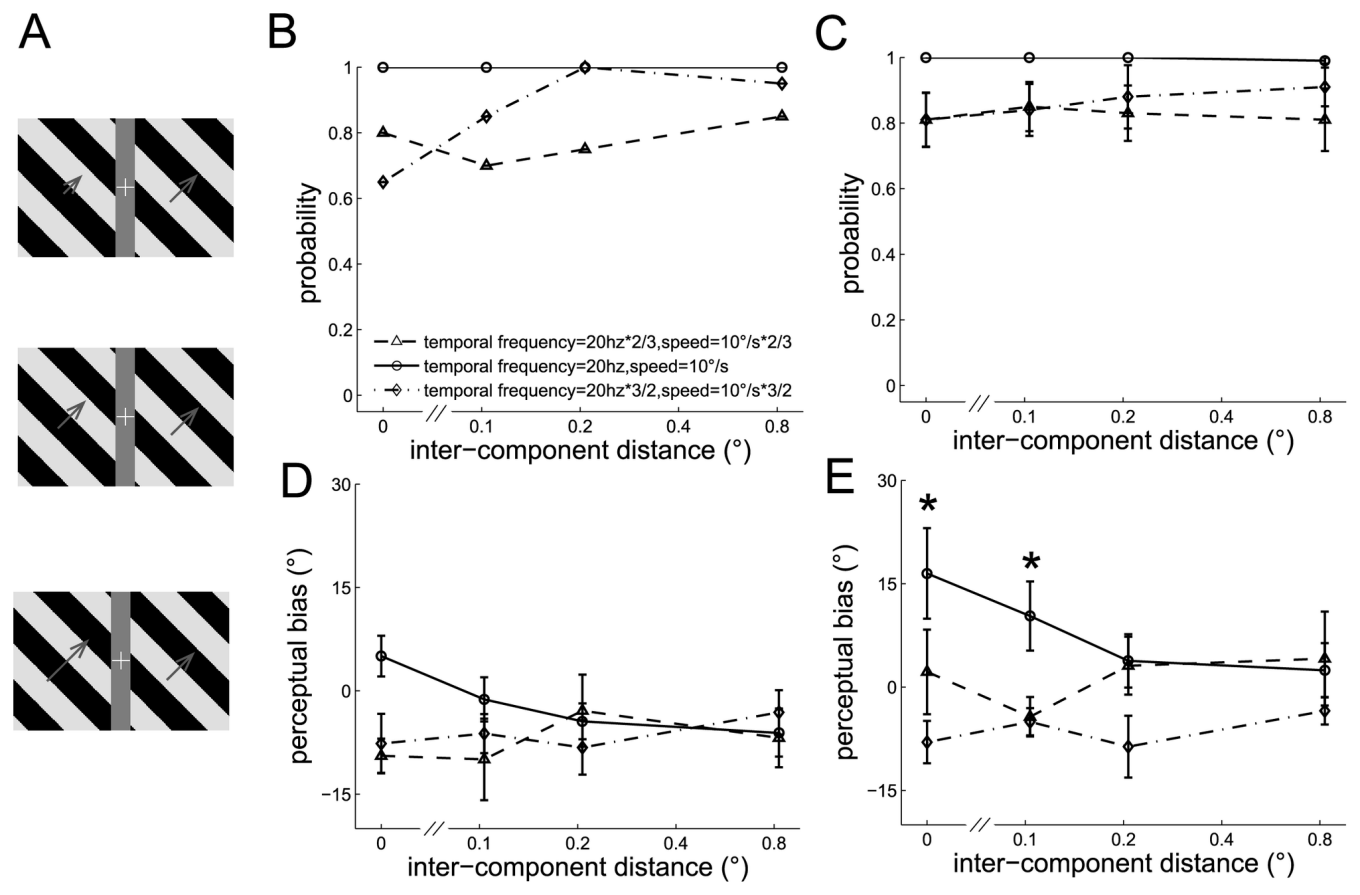
The perceived directions of the dual barber poles in which the temporal frequency of the two component barber poles differed. A. Three example dual barber poles with the temporal frequency of left component barber pole 2/3 times (upper panel), equal (middle panel), or 3/2 times (lower panel) that of the right component barber pole. B–C. The probability the subject perceived a global percept observed in an example subject (B) and in the mean across subjects (C). D–E. The perceptual bias observed in an example subject (D) and in the mean across subjects (E). *: *p* < 0.05 across conditions.

Dual barber poles in which the temporal frequency and corresponding speed in the component barber poles differs tend to elicit a global percept (global probability all > 0.9) (example subject in Figure 5B and the mean across subjects in Figure 5C). The perceptual bias weakened when the temporal frequency differed between the component barber poles (temporal frequency main effect, F(2,8) = 4.6, *p* = 0.048; interaction effect between temporal frequency and inter-component distance, F(6,24) = 4.31, *p* < 0.01). Post-hoc analysis showed that the perceptual bias decreased when the temporal frequency differed in the component barber poles and the inter-component distance was small (*p* < 0.05 when the inter-component distance was 0° and 0.1°) (example subject in Figure 5D and the mean across subjects in Figure 5E), indicating that cross-hemifield integration is weakened by the discrepancy in temporal frequency between the component barber poles even though their wavelength is identical. The discrepancy in temporal frequency did not affect the perceptual bias when the inter-component distance was long (*p* > 0.05 when the inter-component distance was 0.2° and 0.8°).

#### Experiment 6: Discrepancies in wavelength and temporal frequency between the component barber poles while their speed remained the same

**Figure 6:**
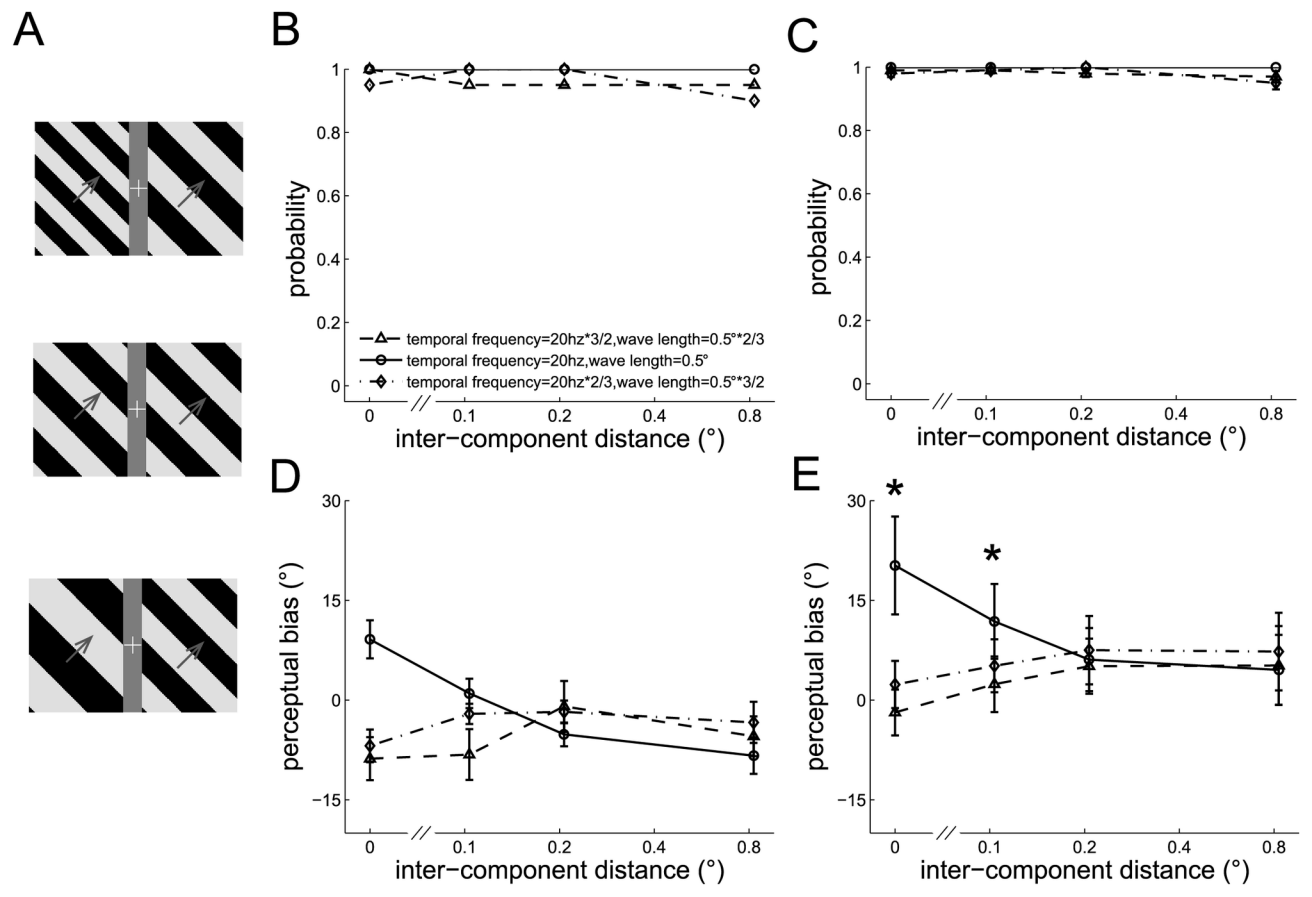
The perceived directions of the dual barber poles in which the two component barber poles had different temporal frequencies, but the same speed. A. Three example dual barber poles with the wavelength of the left component barber-pole 2/3 times (upper panel), equal (middle panel), or 3/2 times (lower panel) that of the right component barber pole. Their speed was fixed so that their temporal frequency ratio was inversely proportional to their wavelength ratio. B–C. The probability the subject perceived a global percept observed in an example subject (B) and in the mean across subjects (C). D–E. The perceptual bias observed in an example subject (D) and in the mean across subjects (E). *: *p* < 0.05 across conditions.

The results in Experiments 4 and 5 indicated that cross-hemifield integration is weakened when the temporal frequency or wavelength differs between the component barber poles. However, speed is the product of wavelength and temporal frequency, and, therefore, any changes in the wavelength (in Experiment 4) or temporal frequency (in Experiment 5) will change the speed proportionally. To determine whether the results in Experiments 4 and 5 could be simply accounted for by the differences in speed between the component barber poles, we varied the wavelength and temporal frequency in the left component barber pole while keeping the speed constant in all conditions. Specifically, the wavelength and temporal frequency of the left component barber pole was 0.33° (0.5°*2/3) & 30 Hz (20 Hz*3/2), 0.5° & 20 Hz, or 0.75° (0.5°*3/2) & 13.3 Hz (30 Hz*2/3) yielding a constant speed of 10°/s (Figure 6A). The wavelength and temporal frequency of the right component barber pole was 0.5° & 20 Hz.

Similar to those observed in Experiments 4 and 5, the dual barber poles whose wavelength and temporal frequency differed in the component barber poles still tended to elicit a global percept (global probability all > 0.9) (See the example subject in Figure 6B and the mean across subjects in Figure 6C). The perceptual bias was weakened when the temporal frequency and wavelength differed between the component barber poles (temporal frequency main effect, F(2,8) = 7.5, *p* = 0.02; the interaction effect between the temporal frequency and inter-component distance, F(6,24) = 5.1, *p* < 0.01). Post-hoc analysis showed that the perceptual bias decreased when the temporal frequency and wavelength differed in the component barber poles and the inter-component distance was small (*p* < 0.05 when the inter-component distance was 0° and 0.1°) (example subject in Figure 6D and the mean across subjects in Figure 6E), indicating that cross-hemifield integration is weakened by discrepancies in wavelength and temporal frequency between the component barber poles even if the speed remains the same. The discrepancies in temporal frequency and wavelength did not affect the perceptual bias when the inter-component distance was long (*p* > 0.05 when the inter-component distance was 0.2° and 0.8°).

#### Experiment 7: Discrepancy in duty cycle between the component barber poles

**Figure 7:**
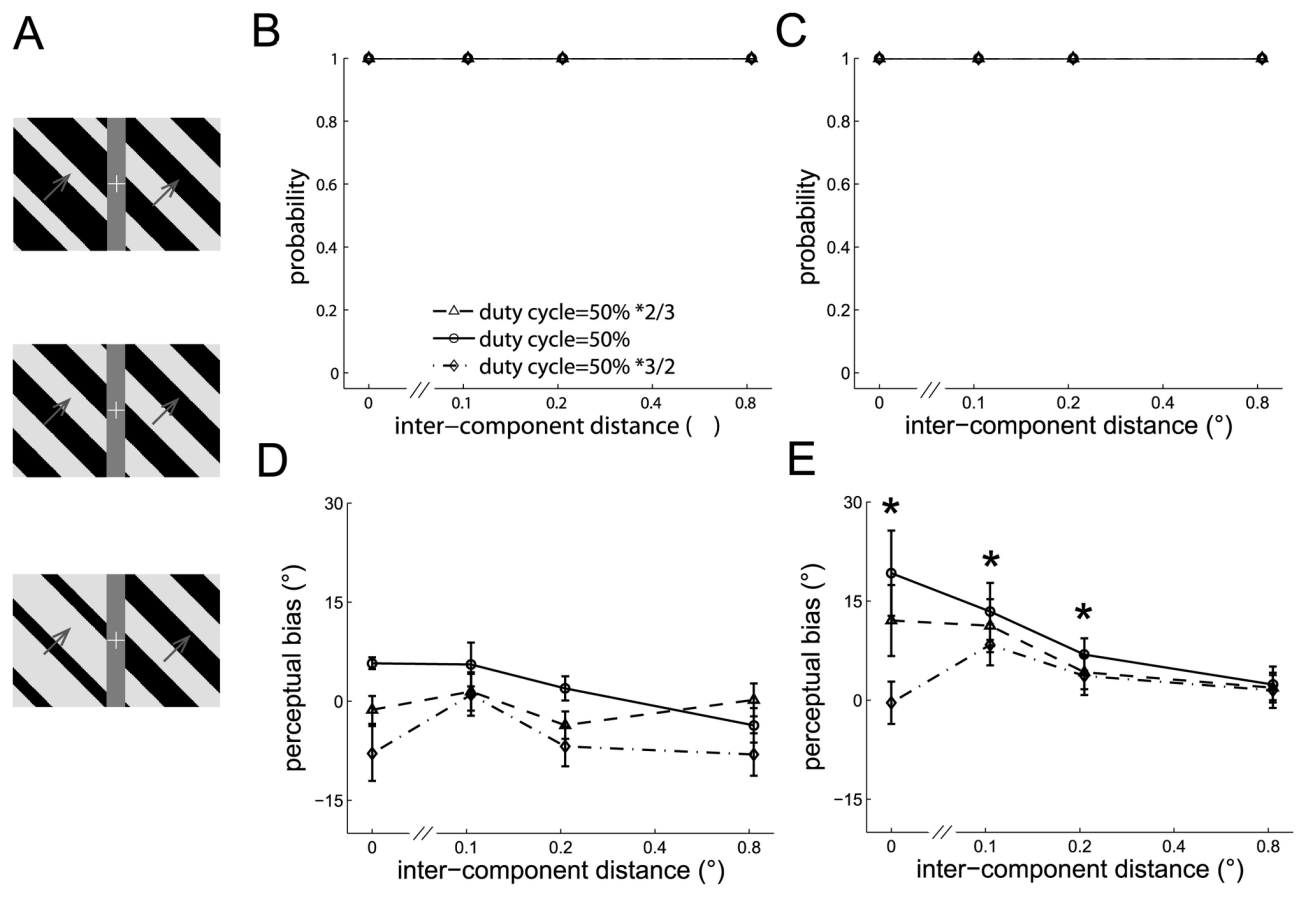
The perceived directions of the dual barber poles in which the duty cycle of the two component barber poles differed. A. Three example dual barber poles with the duty cycle of the left component barber pole 2/3 times (upper panel), equal (middle panel), or 3/2 times (lower panel) that of the right component barber pole (duty cycle = 50%). B–C. The probability of perceiving a global motion observed in an example subject (B) and in the mean across subjects (C). D–E. The perceptual bias observed in an example subject (D) and in the mean across subjects (E). *: *p* < 0.05 across conditions.

We then varied the duty cycle in the left component barber pole while keeping the other parameters the same to test whether a difference in the duty cycle between the component barber poles affects the strength of cross-hemifield integration. The duty cycle of the left component barber pole was 33.3% (50% *2/3), 50%, or 75% (50% *3/2) and that of the right component barber pole was 50% (Figure 7A).

Dual barber poles in which the duty cycle differed in the two component barber poles tended to elicit a global percept (global probability all > 0.99) (See the example subject in Figure 7B and the mean across subjects in Figure 7C). The perceptual bias was weakened when the duty cycle differed between the component barber poles (duty cycle main effect, F(2,8) = 9.6, *p* < 0.01; interaction effect between the duty cycle and inter-component distance, F(6,24) = 3.1, *p* = 0.02). Post-hoc analysis showed that the perceptual bias decreased when the duty cycle differed in the component barber poles and the inter-component distance was small (*p* < 0.05 when the inter-component distance was 0°, 0.1°) (example subject in Figure 7D and mean across subjects in Figure 7E), indicating that cross-hemifield integration is weakened by the difference in duty cycle between the component barber poles. The difference in duty cycle did not affect the perceptual bias when the inter-component distance was long (*p* > 0.05 when the inter-component distance was 0.8°).

#### Experiment 8: Phase difference between the component barber poles

**Figure 8:**
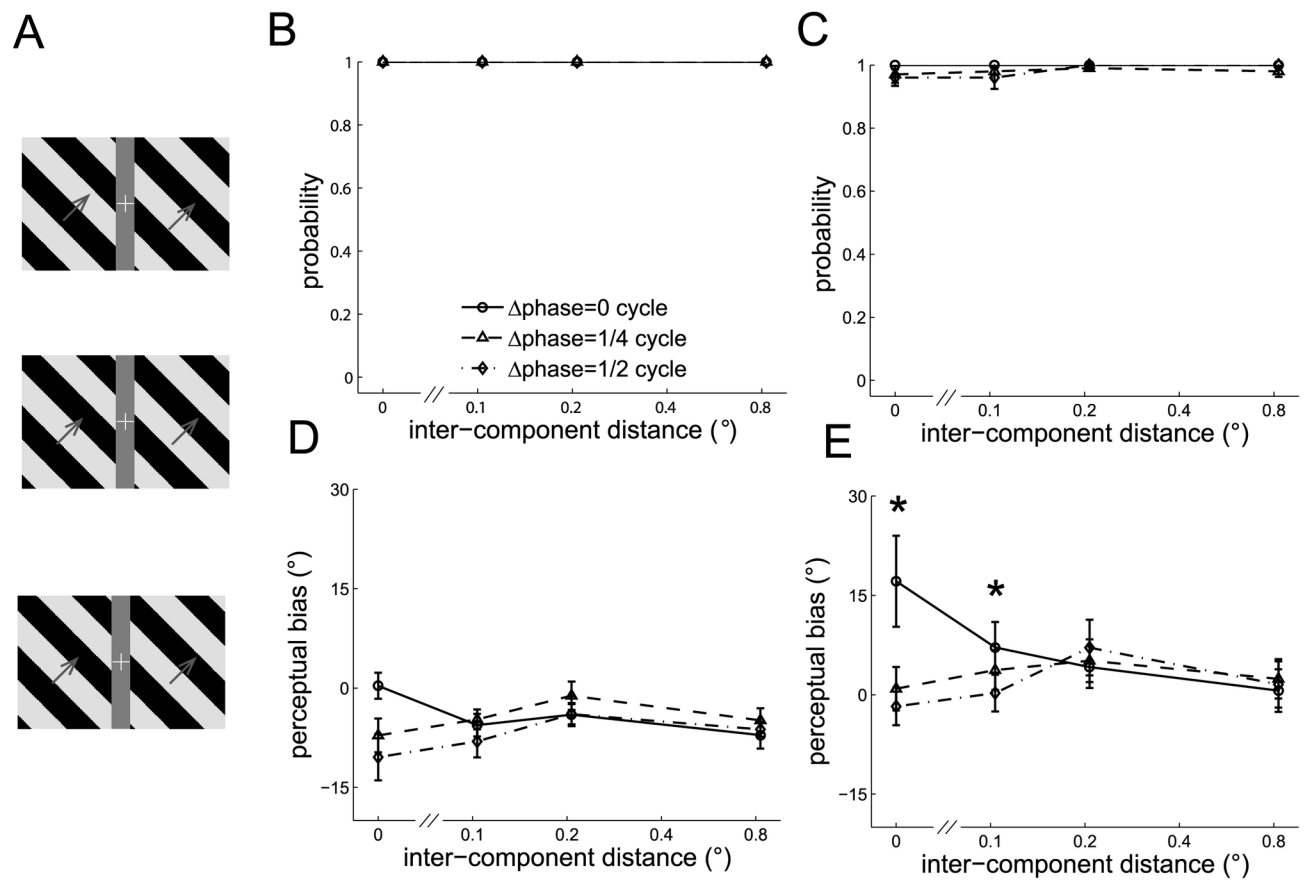
The perceived directions of dual barber poles in which the phase of the two component barber poles differed. A. Three example dual barber poles in which the phase difference between the bilateral component barber poles was 0 (upper panel), 1/4 (middle panel), or 1/2 (lower panel) cycles. B–C. The probability of perceiving a global motion observed in an example subject (B) and in the mean across subjects (C). D–E. The perceptual bias observed in an example subject (D) and in the mean across subjects (E). *: *p* < 0.05 across conditions.

We varied the phase difference between the two component barber poles to measure the effect of phase difference on the strength of cross-hemifield integration. The dual barber poles were presented with phase differences of 0, 0.25, or 0.5 cycles between the two component barber poles (Figure 8A).

Dual barber poles in which the phase differed in the two component barber poles tended to elicit a global percept (global probability all > 0.95) (See the example subject in Figure 8B and the mean across subjects in Figure 8C). The perceptual bias was weakened when the duty cycle differed between the component barber poles (phase difference main effect, F(2,8) = 12, *p* < 0.01; interaction effect between the phase difference and the inter-component distance, F(6,24) = 10.8, *p* < 0.001). Post-hoc analysis showed that the perceptual bias decreased when the phase differed in the component barber poles and the inter-component distance was small (*p* < 0.05 when the inter-component distance was 0° and 0.1°) (example subject in Figure 8D and mean across subjects in Figure 8E), indicating that cross-hemifield integration is weakened by a phase difference between the component barber poles. The phase difference did not affect the perceptual bias when the inter-component distance was long (*p* > 0.05 when the inter-component distance was 0.2° and 0.8°).

#### Experiment 9: Contrast discrepancy between the component barber poles

**Figure 9:**
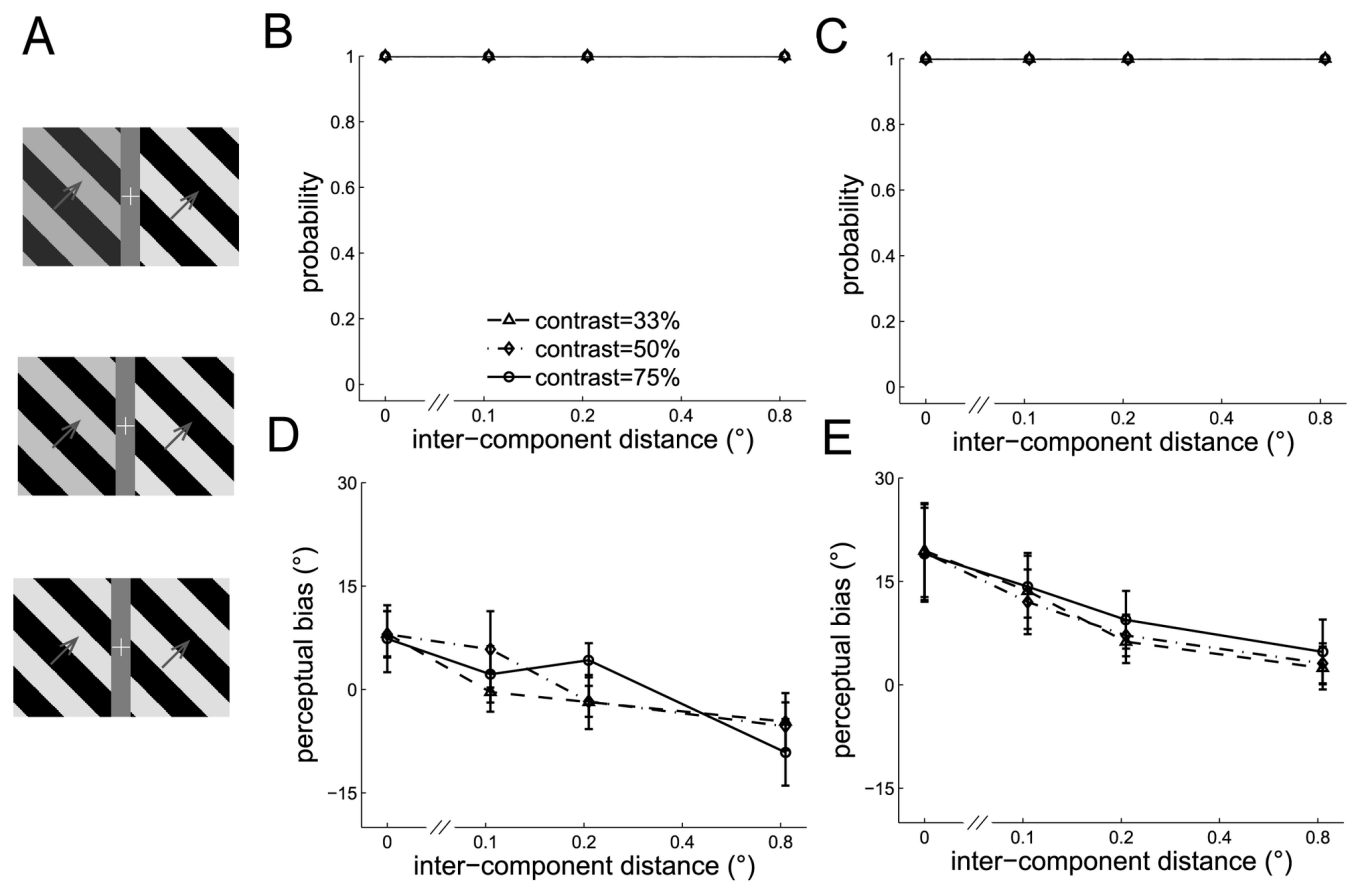
The perceived directions of the dual barber poles in which the contrast of the two component barber poles differed. A. Three example dual barber poles in which the contrast of the left component barber pole was 33% (upper panel), 50% (middle panel), or 75% (lower panel), and the right component barber pole was 75%. B–C. The probability of perceiving a global motion observed in an example subject (B) and in the mean across subjects (C) indicated that the dual barber poles in which the duty cycle differed in the component barber poles could still elicit a global percept. D–E. Surprisingly, the discrepancy in the contrast between the component barber poles did not alter the perceptual bias observed in an example subject (D) and in the mean across subjects (E) (*p* > 0.05 in all cross-condition comparisons).

Finally, we varied the contrast in the left component barber pole while keeping that in the right component barber pole constant to measure the effect of contrast difference between the component barber poles on the strength of cross-hemifield integration. The contrasts for the left component barber pole were 33%, 50%, or 75% and for the right component barber pole was 75% (Figure 9A).

Dual barber poles in which the contrast differs substantially could elicit a global percept (global probability all > 0.99) (See the example subject in Figure 9B and the mean across subjects in Figure 9C). Surprisingly, the perceptual bias did not change when the contrast differed in the component barber poles (contract main effect, F(2,8) = 1, *p* = 0.41; interaction effect between the contrast and inter-component distance, F(6,24) = 0.7, *p* = 0.65), (see the example subject in Figure 9D and the mean across subjects in Figure 9E), a finding indicating the tolerance of contrast difference in the processing of cross-hemifield integration.

## Discussion

A common problem faced by neural systems is integrating information distributed across a space to infer a global stimulus percept. A well-studied example of such an integration problem is the aperture problem [14–17], in which the direction of motion of one-dimensional edges is ambiguous because information about the motion component parallel to their orientation is not available. (This sentence seems to be redundant.) Hence, integrating motion information across the visual image is necessary to recover the veridical velocity of the object by integrating stimulus contours that differ in orientation [24] or by relying on motion signals emanating from terminators (i.e., corners and intersections) whose velocity is unambiguous [9,37].

In the present study, we showed that the visual system tends to integrate motion signals across hemifields. This integration can be achieved using a simple concatenation mechanism when the inter-component distance is short. When the two component barber poles are far apart, the strength of integration is substantially reduced. We first proposed two candidate hypotheses underlying cross-hemifield integration, including the filling-the-gap and simple concatenation hypotheses, which represent the dichotomy of integration complexity. The filling-the-gap hypothesis is compatible with the amodal completion proposed by Gestalt psychology [3,52]. Specifically, the barber pole contours, including the grating edges and corners which do not exist in the visual image, are completed by the visual system. This hypothesis of amodal completion further implies that the barber pole is perceived as being presented behind a foreground aperture [9] and could be achieved by figure ground segmentation mainly carried out by neurons in visual cortex V2 [53–55]. On the other hand, the simple concatenation hypothesis, which is supported by the results in the present study, is compatible with the vector average model proposed by Pack et al. [38]. Based on this model, the perceived direction is biased toward the long axis because the long axis of the aperture is comprised of more terminators than the short axis. In conditions with longer inter-component distances, the visual system is less likely to regard component barber poles as parts of a global object so the strength of integration is weakened. We hypothesize that these integration properties are mediated by neurons in the middle temporal (MT) cortex, whose receptive fields are relatively large (~10°) [56,57] and can cover stimulus presented in both hemifields (< 3°). The neural correlate of illusionary motion has also been located in V5/MT in neurophysiological [58] and transcranial magnetic stimulation studies [59]. Also, the neural signals have been shown to reflect the magnitude of barber pole illusion in several barber pole variants [38,50]. As the cross-hemifield integration is most robust when the stimulus size is limited to within 3°, it is less likely to be mediated by the property of integrative non-classical receptive field of MT neurons which is recently proposed by Huang et al. [60,61]. Another interesting finding of the present study is that the fulfillment of a series of contour and motion constraints, including wavelength, temporal frequency, speed, duty cycle, and phase, is necessary for successful cross-hemifield motion integration. Also, the influence of these constraints is most robust when the two component barber poles are placed close to each other. Accordingly, we hypothesize that these constraints will affect the continuity of the edges of the square-wave gratings across the component barber poles, since contour continuity has been shown to determine the strength of perceptual grouping [62,63]. It appears that the continuity of grating edges across apertures is the most fundamental determinant of integration. In other words, any offset of the grating edges across apertures will weaken the strength of integration. The fact that cross-hemifield integration remains strong when the contrast of the component barber poles differs substantially indicates a strong tolerance of contrast difference across apertures. As transparency, reflection, and shadowing usually cause a discrepancy of luminance across apertures, it is reasonable that the visual system develops a strategy for resolving the contrast difference across the apertures. Interestingly, the contrast tolerance is compatible with the aforementioned edge continuity rule, as the change of contrast induces no offset of grating edges across apertures.

In the present study, we chose the barber pole illusion to study how local motion signals are integrated to yield a global motion percept. Indeed, the mechanisms of motion integration can be characterized by several other visual illusions: The Pinna-Brelstaff illusion [64–66] demonstrated that local motion signals are processed by spatiotemporally tuned, orientation sensitive units in area V1. The rotating tilted lines illusion [26,67,68] revealed that contour neurons can be weighted more than end-stopped neurons in determining the perceived direction of motion. The dancing bar illusion [69] showed that visual motion perception is not determined by the internal terminators when a bar is oscillating at certain frequencies. The accordion grating illusion [28,29] illustrated a new three-dimensional aperture problem incorporating depth signals. The unchained dots illusion [70] reflected the perceptual compromise between apparent motion and veridical motion.

Shimojo et al. first presented the cross-aperture barber pole illusion by demonstrating that a grating stereoscopically seen through three horizontal square apertures (arranged vertically) on an occluding surface is perceived as moving vertically. This barber pole illusion greatly diminished when the gratings in the rectangular apertures were placed in front of the occlusion plane, indicating that motion integration across apertures occurs only when the grating plane is behind the occlusion plane [9]. In the present study, we further illustrated that motion integration across apertures can also work in a two-dimensional setup in which the component barber poles and background are on the same frontal-parallel plane. The terminators, such as the intersections between the grating edges and aperture boundary of the barber poles created in the present study, are fundamentally different to those in Shimojo’s study: the former setup yields intrinsic terminators while the latter yields extrinsic terminators. As intrinsic and extrinsic terminators have shown distinctively different properties in the determination of direction of barber pole motion [38,46–50], future studies are needed to address how the visual system uses both the depth and terminator cues to infer the direction of motion of an object seen through multiple apertures.
